# Tuberculosis Outbreak in a Primary School, Milan, Italy

**DOI:** 10.3201/eid1902.120527

**Published:** 2013-03

**Authors:** Marino Faccini, Luigi Ruffo Codecasa, Giorgio Ciconali, Serafina Cammarata, Catia Rosanna Borriello, Costanza De Gioia, Alessandro Za, Andrea Filippo Marino, Valentina Vighi, Maurizio Ferrarese, Giovanni Gesu, Ester Mazzola, Silvana Castaldi

**Affiliations:** Author affiliations: Prevention Department ASL Milano, Milan, Italy (M. Faccini, G. Ciconali, S. Cammarata, C.R. Borriello, C. De Gioia);; Azienda Ospedaliera Ospedale Niguarda Cà Granda, Milan (L.R. Codecasa, M. Ferrarese, G. Gesu, E. Mazzola);; University of Milan Postgraduate School of Public Health, Milan (A. Za, A.F. Marino, V. Vighi, S. Castaldi);; Fondazione IRCCS Ca’ Granda Ospedale Maggiore, Milan (S. Castaldi)

**Keywords:** outbreak, tuberculosis, school, children, genotyping, tuberculosis and other mycobacteria, bacteria, Italy, Milan, diagnosis, disease management, control, surveillance, homeless, Beijing strain, epidemiology

## Abstract

Investigation of an outbreak of tuberculosis (TB) in a primary school in Milan, Italy, found 15 schoolchildren had active TB disease and 173 had latent TB infection. TB was also identified in 2 homeless men near the school. Diagnostic delay, particularly in the index case-patient, contributed to the transmission of infection.

Italy has a low incidence of tuberculosis (TB); in 2008, incidence of notified cases was 7.6/100,000 population (*1*). However, higher incidence rates have been reported in some areas. In 2009, in the northern Italy region of Lombardy, incidence of notified cases was 11.7/100,000 population; 58% of cases were in non-Italian nationals (www.dgsan.lombardia.it/malinf/2009/report_sintesi_2009.pdf). Incidence in children 0–14 years of age was 3.38/100,000 (n = 47 cases). In 2009 in Milan, the largest urban area of Lombardy (1.6 million inhabitants), the incidence was 20.44/100,000 population (www.asl.milano.it/user/download.aspx?FILE=OBJ06171.PDF&TIPO=FLE&NOME=report_prevenzione_2011).

In industrialized countries, such as Italy, TB is increasingly associated with specific population subgroups: immigrants from countries with high endemicity (*2**,**5**,**6*), ethnic minorities (*2*), refugees, and the homeless (*2*,*4*). The control of TB in Italy relies on timely diagnosis and adequate treatment of TB cases, screening of persons in at-risk groups and those in close contact with active TB case-patients, and vaccination of at-risk health care workers and children who live in close contact with a reported TB case-patient (*2*,*3*). Factors that influence the effectiveness of TB surveillance and control include lack of prioritization of TB within the health service, difficulties faced by foreign citizens in accessing health care, lack of coordination by a reference center, and diagnostic delay (*7*). One study found that that median diagnostic delay, health care delay, and total delay for TB patients in Italy were 7, 36, and 65 days, respectively (*8*). We investigated an outbreak of TB infection identified in 2010 among children in a primary school in Milan.

## The Study

In November 2010, pulmonary and meningeal TB was identified in a 7-year-old boy in Milan who was in the second year of primary school. The child experienced fever, headache, and asthenia in September 2010 and was treated by a pediatrician; no radiographs were taken. Two months later, the child was hospitalized because the severity of symptoms had increased, and Beijing strain *Mycobacterium tuberculosis* bacteria were isolated from a gastric lavage sample.

As a result of this case, local health authorities conducted a contact investigation. Screening of potential contacts is done by using the Mantoux tuberculin skin test (TST); induration of >5 mm is considered positive for this test (*9*). Because a delay of 8–10 weeks after exposure is necessary for a positive skin test, if a contact’s exposure to the TB case-patient occurs within that period, TST is repeated 8–10 weeks after the most recent exposure. Persons who have received bacille Calmette-Guerin vaccine and have positive TST results are subjected to the interferon-gamma release assay test. Those with positive results for either test undergo clinical evaluation and chest radiography. If a diagnosis of active TB is ruled out, the person is defined with a case of latent TB infection (LTBI).

In this investigation, the boy’s family and friends all had negative TST results. His classmates and the children in the 2 adjacent classrooms were screened; results indicated LTBI for 20% of the students in his classroom and 20% and 14% of students in the other 2 classrooms. His teachers and some other school staff members were also screened (n = 43); no cases of active TB were detected among children or school staff.

In December 2010, pleural TB was confirmed by isolation of *M. tuberculosis* from a secondary school pupil who had attended the same primary school as the first case-patient during the previous year. Genotyping confirmed that the isolate was the same Bejing strain. A search of the regional strain database found that this genotype had also been identified in a case of pulmonary TB reported in November 2009 in a homeless person who lived in the city park in front of the school. This man had been lost to therapeutic follow-up but had infected his daughter.

After the second school case was identified, health authorities extended TST testing to all children who had attended the school during 2010, including those who had moved to other schools (Table), and to all school staff members. Thirteen homeless persons who frequented the area around the school were also screened; 1 was found to have TB, and the *M. tuberculosis* isolated was of the same Bejing strain as that isolated from the other homeless man and the first 2 infected schoolchildren.

**Table:** Distribution of prevalence rates of TB and LTBI among current and former students at primary school, Milan, Italy, 2011*

Class	No. students	No. (%) undergoing TST	No. (%) TST positive†			Total no. (%) active TB cases‡
			First test	Second and third tests	Total positive	
First year	162	161 (99.4)	3 (1.9)	0	3 (1.9)	1 (0.6)
Second year	174	172 (98.9)	47 (27.3)	3 (1.7)	50 (29.1)	5 (2.9)
Third year	170	170 (100)	11 (6.5)	0	11 (6.5)	0
Fourth year	156	156 (100)	30 (19.2)	3 (1.9)	33 (21.2)	2 (1.3)
Fifth year	151	149 (98.7)	57 (38.3)	2 (1.3)	59 (39.6)	4 (2.7)
Former fifth year§	156	156 (100)	29 (18.6)	ND	29 (18.6)	3 (1.9)
Transferred	20	13 (65)	3 (23.1)	ND	3 (23.1)	0
Total	989	977 (98.8)	180 (18.4)	8 (0.8)	188 (19.2)	15 (1.5)

*Because a delay of 8–10 weeks after exposure is necessary for a positive skin test, follow-up screening was performed for students whose initial test occurred <10 weeks after exposure. TB, tuberculosis; LTBI, latent TB infection; TST, tuberculin skin test; ND, not done because initial screening was >10 weeks after exposure. †At the first screening, 3 students refused to submit to TST; 11 refused at the second, and 11 at the third. ‡All other infections were LTBI. §Those who were fifth-year students during the previous school year.

A total of 15 cases of TB were identified among current and former students at the school, including 2 pulmonary forms, 1 extrapulmonary form confirmed bacteriologically, and 12 early pulmonary forms without *M. tuberculosis* isolation. The latter type is defined as clinical evidence of the disease and any of the following: contact with an adult with TB, positive TST results, suggestive appearances for TB on chest x-ray, and favorable response to antituberculous therapy (*10*). A total of 173 pupils were found to have LTBI. No active TB was found among the school’s staff members.

**Figure 1:**
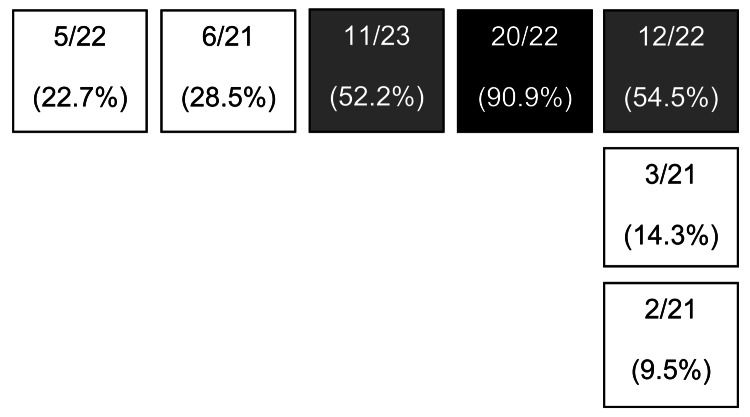
Floor plan of classrooms at primary school in Milan, Italy, 2010. Values are no. tuberculosis infections/total no. pupils in classroom (% pupils infected). Shading indicates classrooms with highest rates of infection: the classroom of the 17-year-old student determined to be the index case-patient (black shading) and the 2 adjacent classrooms (gray shading).

In February 2011, another case of pulmonary TB was reported. The patient, a 17-year-old boy who had been coughing for ≈1 month, was in the fifth year of primary school with younger children because of disabilities (spastic tetraplegia). He had been in the primary school for 3 years. He had the same *M. tuberculosis* genotype (isolate from gastric lavage) as found in the previous cases. The collected data suggest that this boy was the index case-patient for this outbreak: 90.9% of his classmates were infected, and a relevant ratio of TB infection was found among pupils of the other classrooms on the same floor (Figure 1). The spread of infection was probably favored by the fact that, because of his disability, this student was part of an integration program that included taking part in activities in other classes and areas of the school. In addition, positive TST results among former fifth-year primary pupils (in the first year of secondary school at the time of testing) show that the disease was already active during the previous school year.

**Figure 2:**
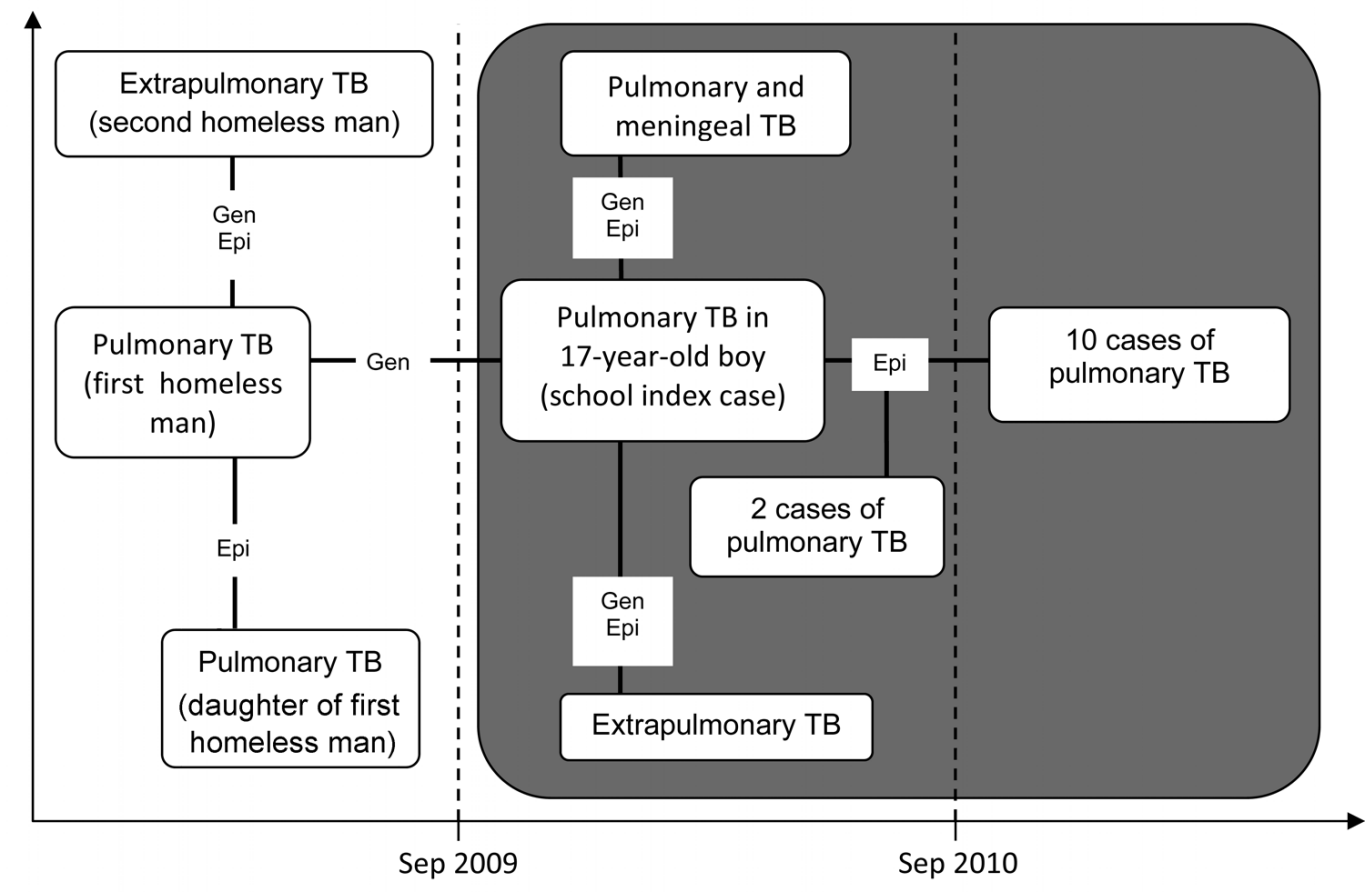
Hypothetical timeline of tuberculosis (TB) outbreak at primary school in Milan, Italy, 2009–2011. Vertical dashed lines indicate school year delineation. The connection among cases established through epidemiologic investigation (epi) or genotyping of *Mycobacterium tuberculosis* strains (gen) is indicated.

Genotyping has proved to be an essential tool in TB contact investigations (*11*), especially in the presence of clusters. Genotyping of *M. tuberculosis* isolates during this investigation enabled the establishment of a connection between the cases of TB among the schoolchildren. However, no contact between the 17-year-old boy and the first homeless man has been identified (Figure 2).

## Conclusions

The results of this investigation indicate that a diagnostic delay for the index case-patient played a primary role in the transmission of infection inside the school. The main cause of this delay was the low degree of diagnostic suspicion toward the disease; however, TB can also be difficult to diagnose in children because children are less able to produce sputum. Physicians should be aware of the signs and symptoms of early TB infection and should consider this diagnosis accordingly. Furthermore, routine screening for TB could be considered for persons with disabilities or special needs who take part in recreational and educational activities in which they come into close contact with more susceptible groups, such as children.

In addition, cases of TB among the homeless, particularly those in close proximity to susceptible groups such as schoolchildren, highlight the problem of therapeutic monitoring among persons who may be lost to follow-up. Careful evaluation of compliance at time of discharge from health care is critical, and social protection programs are needed to improve rates of follow-up care. In particular, in urban areas where risk factors for transmission of TB are highly concentrated, a TB reference center may improve collaboration between local health authority, physicians, and social services.
